# Mandibular bone mass density in a medieval population and its relationship with stable isotopes δ13C and δ15N

**DOI:** 10.1007/s10266-024-00968-4

**Published:** 2024-07-06

**Authors:** Concepción López-Leyva, Silvia Jiménez-Brobeil, Antonio Magán-Fernández, Cristina Benavides-Reyes, Manuel Bravo, Francisco Mesa

**Affiliations:** 1 Department of Periodontics, School of Dentistry, University of Granada, Granada, Spain; 2 Department of Legal Medicine, Toxicology and Physical Anthropology, University of Granada, Granada, Spain; 3 Department of Operative Dentistry, School of Dentistry, University of Granada, Granada, Spain; 4 Department of Preventive and Community Dentistry, University of Granada, Granada, Spain

**Keywords:** Bone density, Radiometric indexes, Isotopes, Panoramic radiography, δ15N

## Abstract

The aim of this study was to compare the level of bone mass in digital orthopantomograms in two populations (medieval and current) using two radiomorphometric indexes, and to correlate the mandibular bone mass value, in the medieval mandible population, with stable isotope data δ13C and δ15N. An observational, cross-sectional, and analytical study on mandibles from two diachronic groups, 15 mandibles from the medieval settlement of La Torrecilla (Granada, Spain) and 15 mandibles from current patients at the Faculty of Dentistry of the University of Granada (Spain), matched by age and sex was conducted. The bone mass density was determined using the Mandibular Cortical Width Index (MCW) and the Mandibular Panoramic Index (PMI) in digital panoramic radiographs. In the medieval group, the values of bone mass density were correlated with those of two stable isotopes (δ13C and δ15N). The mean value of MCW in mm in the medieval group was 3.96 ± 0.60 (mean ± standard deviation) and in the current group was 4.02 ± 1.01. The PMI was 0.33 ± 0.06 and 0.35 ± 0.08 in the medieval and current groups respectively, with similar results in both groups (*p* = 0.820 and *p* = 0.575). A negative correlation was found between both morphometric indices and the δ15N isotope (rs = 0.56, *p* = 0.030 and rs = 0.61, *p* = 0.016, respectively). The bone mass density in mandibles belonging to the two compared populations, determined by two quantitative radiomorphometric indices, is similar. Within the medieval population, there is an inverse correlation between the δ15N value and bone mass density.

## Introduction

A better understanding of past populations is of interest from anthropological, medical, and dental perspectives. The Nasrid kingdom (1232–1492) occupied an area of about 20,000 km^2^ in southern Spain [1]. The “alquería” is the settlement of rural Muslim communities [2]. Guinchar (1987) defined it as “a small rural community formed by some tens of houses, homes, or families in general, who exploited a piece of land without social or economic dependence on a feudal lord.” They had irrigation areas for cultivation and self-sufficiency and areas for grazing, hunting, collecting firewood, etc. They had a mosque, an oven, and a cemetery outside the village. The houses were organized in blocks, not always open to the street, according to Muslim custom. The Islamic necropolis of the La Torrecilla alquería is located in the region of Alhama de Granada, about 60 km from the city of Granada, and was excavated between 1968 and 1979 [3, 4]. One hundred and fifty two tombs belonging to a rural population were found, with a similar number of male and female [5], placed following Islamic ritual. Several fragments of ceramic material were found in one of the tombs, two of which corresponded to the same vessel, made of unglazed ochre paste with incised lines that could chronologically belong to the late 13th or early fourteenth century.

Reconstructing the diet of ancient populations (paleodiets) from bone remains (bone collagen) and teeth (dentin) has been used in archeology since the mid-1970s. For this purpose, the determination of the isotopic deviation (δ) of stable isotopes, which not decay over time, mainly carbon (12C/13C) and nitrogen (14N/15N), is used [6]. Based on the principle that human nutrition is recorded in organic tissues according to a predictable isotopic fractionation [7], this determination, using a mass spectrometer, indicates how far the sample deviates from a previously defined international pattern (marine fossil carbon and atmospheric nitrogen, respectively) [8, 9].

The mandible is a bone structure highly sensitive to changes in body bone mass. Numerous studies have demonstrated the correlation between mandibular bone density and the density of postcranial bones (especially the spine and femur) [10]. Several radiomorphometric indexes based on the measurement of mandibular anatomical structures in panoramic radiographs have been published, and their values are correlated with bone mineral density (BMD) [11–13]. The use of orthopantomograms to measure radiomorphometric indexes offers an adequate assessment of BMD and therefore the risk of osteoporosis, using a simple and inexpensive method [14]. In addition, our research group has experience with the use of this methodology for BMD analysis [9].

The objective of this study was: (1) to compare the level of bone mass in two populations (medieval and current) in digital orthopantomograms using two radiomorphometric indexes, and (2) to correlate the mandibular bone mass value in the medieval mandible population, with stable isotope data δ13C and δ15N.

## Materials and methods

### Study design and population type

An observational, cross-sectional, and analytical study on mandibles from two diachronic groups was conducted. On the one hand, 15 mandibles belonging to the medieval necropolis of “La Torrecilla” were analyzed. The chronology of this cemetery ranges from the 13th to the fifteenth century AD according to recent ^14^C datings [15]. The skeletal remains were deposited in the Anthropology Department of the Faculty of Medicine, University of Granada (Spain).

For the estimation of sex of the individuals, the morphology of the pelvis and skull were considered. Age estimation was performed by intervals based on changes in the pubic symphysis, auricular surface of the ilium, and sternal end of the ribs as described in [16]. Subsequently, current patients' mandibles were matched by these two variables (sex, age).

The corresponding data of δ13C and δ15N isotopes were provided by the Department of Anthropology at the Faculty of Medicine (University of Granada, Spain). Collagen from rib remains that present a turnover of approximately 5 years was extracted for analysis. The methodology for the quantification of isotopes is described in a previous article by one of the authors [17].

On the other hand, the current group consisted of 15 orthopantomographies of actual individuals, stored in the database of the Radiology Service of the Faculty of Dentistry, University of Granada (Spain). Since these are archeological remains and radiographs from a database, the study does not require ethical evaluation.

### Radiological and sociodemographic variables

The 15 mandibles from the medieval group were digitized under the same conditions. All panoramic radiographs were taken by the same technician using the same orthopantomograph (Planmeca, Promax 3d, Helsinki, Finland) with a magnification of 1.2 and an exposure value of 62 kV of 5 mA, 15.8 s for both sexes.

**Fig. 1:**
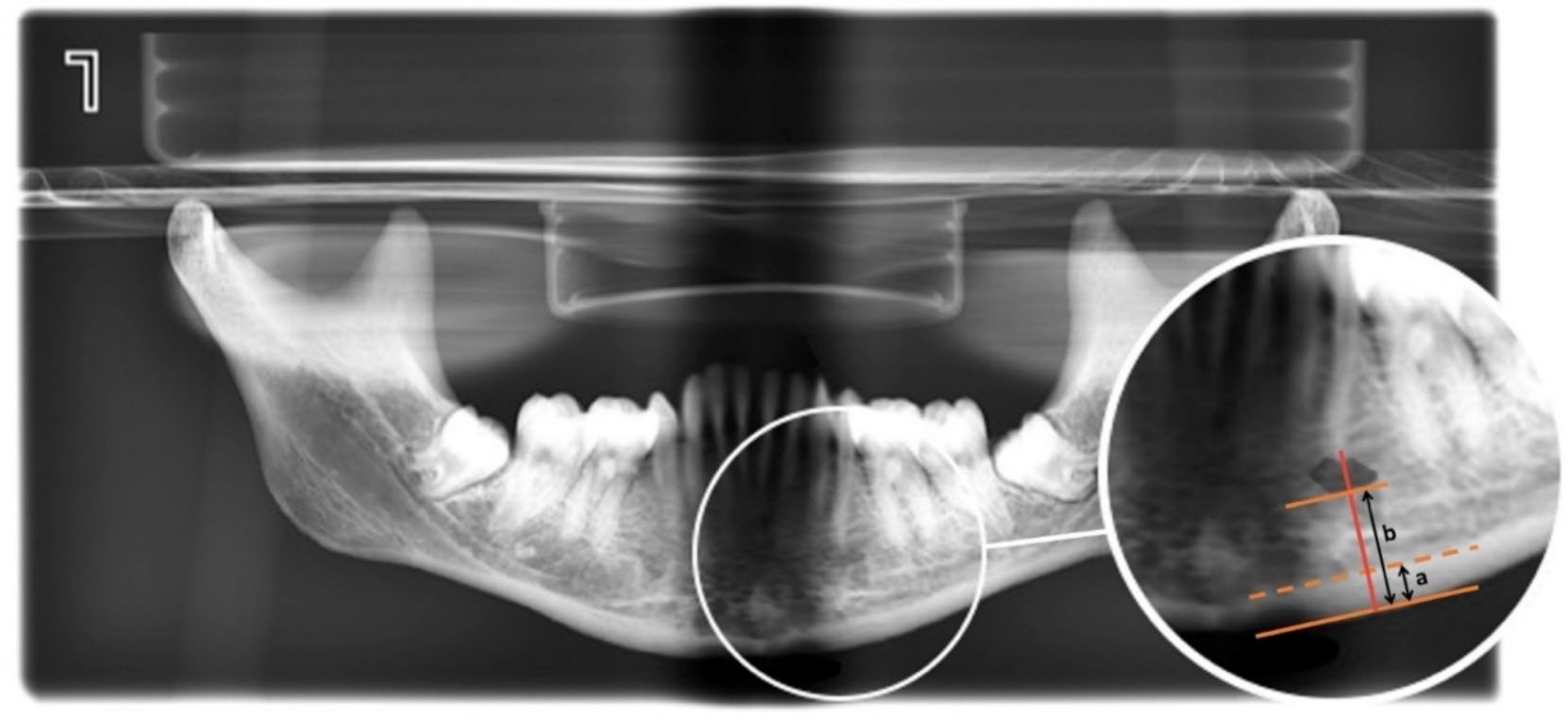
Own elaboration on a mandible from the La Torrecilla necropolis. MCW: Taguchi Mandibular Cortical Width Index (a). PMI: Benson Index (a/b)

The radiomorphometric indices of [11, 13] were determined on the radiographs. To do this, two dentists (C.L. and F.M) located the anatomical structures that were measured. The Taguchi index reflects the width of the mandibular cortical bone at the intersection between a line that divides the mental foramen and is tangential to the mandibular inferior border. On the other hand, the Benson Mandibular Panoramic Index is calculated by dividing the thickness of the mandibular cortical bone, located at the mental foramen, by the distance from the lower margin of the mandible to the lower edge of the foramen (Fig. 1).

Researcher CL was calibrated by intra-examiner reproducibility analysis. For this purpose, 10 radiographs were selected and measured with a 2-week interval. The intraclass correlation coefficient was 0.881, indicating good agreement.

The measurements were made by capturing the radiographic image with the digital image analysis program Image J®. Prior to measurement, the program was calibrated in mm.

### Statistical analysis

Statistical analysis was performed using EPIINFO 5.0 and SPSS 20.0, with methods explained in the footnotes of each table. The sample size (*n* = 15 per group) allows for the comparison of quantitative variables of effect (DMO) between the two groups with a power of 80%, alpha error of 5%, and to detect a standardized difference of 1.0, which is between Large according to the cutoff of 0.8 on the scale of [18] and Very large according to the cutoff of 1.2 on the scale of [8] The Sample Power 2.0 program (SPSS Inc., Chicago, IL) was used.

## Results

Fifteen digital orthopantomographs of mandibles from a medieval population and 15 from a current population, matched by sex and age, were analyzed. The distribution by age was 10 females (66.7%) and 5 males (33.3%). For the age distribution, given that it cannot be precisely defined in anthropological populations, the mean of an age range was established and compared to the known age of current individuals. Age was thus classified into three age groups: 18 years (1 mandible (6.7%)), 30 years (12 mandibles (80%)), and 50 years (2 mandibles (13.3%)), with a mean age of 32 ± 8 years.

The results of the stable isotope δ13C and δ15N deviation analysis, which was only determined in medieval samples, are shown in Table 1. Some of the samples did not meet the criteria for good collagen quality since the percentage of N is slightly less than 10% and that of C is 30% [19–21], but they do meet the C/N ratio which must be between 2.9 and 3.6.

**Table 1:** Characteristics of the populations analyzed and results of the stable isotope δ13C and δ15N deviation analysis

Variable	Medieval (*n* = 15)	Current (*n* = 15)	*P *value
Sex, *n* (%)			B
Women	10 (66.7)	10 (66.7)	
Men	5 (33.3)	5 (33.3)	
Age			B
18	1 (6.7)	1 (6.7)	
30	12 (80.0)	12 (80.0)	
50	2 (13.3)	2 (13.3)	
Mean ± sd	32 ± 8	32 ± 8	
^13^C (carbon)^a^ Range Mean ± sd	–17.6 a -14.0 –15.7 ± 1.0		
^15^N (nitrogen)^a^ Range Mean ± sd	9.14 a 11.14 9.94 ± 0.53		

^a^Only available in Medieval group. Isotopic deviation (δ)

^b^Note that the two groups are matched for age and sex

The comparison of two radiomorphometric indexes, Taguchi et al., (MCW) and Benson et al., (PMI), showed very similar values in both medieval and current groups (mean ± SD) with 3.96 ± 0.60 and 4.02 ± 1.01 for MCW and 0.33 ± 0.06 and 0.35 ± 0.08 for PMI, respectively. The indicative levels of increased risk of possible diagnosis of osteoporosis have been described as < 3 mm and < 3.92 mm in female and men, respectively, according to MCW, and < 0.3 mm according to PMI. Following this classification, there were only 3 cases in medieval mandibles and 2 in current mandibles according to MCW, and 4 cases in medieval mandibles versus 5 in current mandibles according to PMI, showing no statistically significant differences (Table 2).

**Table 2:** Radiological characterization of the populations analyzed

Variable	Medieval (*n* = 15)	Current (*n* = 15)	*P*value
MCW mm. (Taguchi), Mean ± sd	3.96 ± 0.60	4.02 ± 1.01	0.820^c^
Osteoporosis (Taguchi), mm			≈1^d^
No	12 (80.0)	13 (86.7)	
Yes^a^	3 (20.0)	2 (13.3)	
PMI mm. (Benson), Mean ± sd	0.33 ± 0.06	0.35 ± 0.08	0.575^c^
Osteoporosis (Benson)			≈1^d^
No	11 (73.3)	10 (66.7)	
Yes^b^	4 (26.7)	5 (33.3)	

^a^< 3 mm in women *y* < 3.92 mm in men

^b^< 0.3 mm

^c^Mann–Whitney test

^d^Bilateral Fisher's exact test

Table 3 represents the non-parametric Spearman correlations, where a statistically significant negative correlation was observed between both radiomorphometric indexes and the δ15N isotope. The δ13C was not associated with either of the body mass bone indexes.

**Table 3:** Spearman’s correlation (rs) between bone mass and isotopes in medieval (*n* = 15)

Variable	δ^13^C (carbo)	δ^15^N (Nitrogen)
MCW mm. (Taguchi)	*r*_s_ = 0.17, *p* = 0.554	*r*_s_ = –0.56, *p* = 0.030
PMI mm. (Benson)	*r*_s_ = 0.06, *p* = 0.845	*r*_s_ = –0.61, *p* = 0.016

## Discussion

The two main results of our study were obtaining similar values in BMD determined by two radiomorphometric indices in mandibles between two populations, one medieval and the other current. In addition to similar values, there were only three cases in the medieval samples versus two in the current population with a diagnosis of a higher risk of osteoporosis according to MCW.

The second result was that δ15N levels showed a statistically significant inverse correlation with the degree of bone mass.

To the best of our knowledge, there is no scientific literature on this type of methodology for the determination of BMD applied to osteological populations. However, bone mass loss has been studied in ancient populations using other techniques, mainly densitometry, the gold standard for the diagnosis of osteoporosis [22, 23], in 185 femurs of a medieval Norwegian population, obtained a higher BMD in the rural population compared to a population of monks from a monastery in the area. The authors related it to greater physical activity of the former.

In an ancient population of Southern Patagonia, also showed a reduction in BMD by over 25% in female over 30 years old by densitometry and related it to food deficits, pregnancies, and lactation [24].

On the remains of six elite Moche characters in Peru, showed by densitometry a decreased bone mass density (in the II and VII centuries AD) [25]. The authors' conclusions were that the sedentary lifestyle of these noble members would have more influence on the loss of BMD than their diet.

All these investigations can provide information on the role of extrinsic factors in the appearance of osteopenia and osteoporosis in these past societies. With our study, we demonstrate, with different methodology, how BMD is similar in two populations in southern Spain, separated in time by almost 900 years. Even in the case of a medieval rural population with totally different eating habits and social life, this did not influence a different BMD.

One aspect to consider in archeological bone remains, when comparing their characteristics, would be the problems of correctly assigning the estimation of the subjects’ age. In the case of BMD, the positive correlation between increasing age and bone mass loss is well known [26]. To avoid this bias, we dated the mandibles according to the changes observed in three different types of bones of the skeleton, according to [16], and estimated an age range.

We then matched the age to compare them with current mandibles. Another aspect in our favor is that the measurement of the two radiomorphometric indices, unlike the authors who described them [11, 13] who did it manually with rules and calipers, was carried out using a digital image program (Image J®) that ensures more accurate and reliable results. Our medieval population, sharing the opinion of previous researchers, although from a poor social class, with a deficient diet, could present greater physical activity, which, in terms of bone mass, would compensate for possible nutritional deficiencies compared to the current population, where a more complete diet would balance the excess of sedentary lifestyle characteristic of modern civilization.

Plants use atmospheric CO_2_ (^12^C) in three radically different photosynthesis routes, this implies the appearance of different fractionations in carbon isotopes (^12^C and δ^13^C), which once known, show us the types of plants and the parameters organic ingredients used in each human diet [27]. Therefore, depending on the results for δ^13^C, we will distinguish a diet rich in C_3_ plants from consumers of C_4_ plants that are even more enriched in ^13^C [28]. The C_3_ plant group is represented by plants typical of temperate and cold climates (wheat, barley, rice…), while the C_4_ plants are typical of tropical, savannah and dry climates (millet, corn, sorghum, sugar cane…) [29].

The results obtained in the Nasrid population with respect to this ^13^C isotope show that the diet followed by this community, surely based on carbohydrates, did not influence their BMD, as demonstrated by the total absence of relationship between both variables.

Regarding the isotopic ratio of δ^15^N, higher figures are indicative of the consumption of foods rich in animal proteins, lower values, in principle, would indicate the consumption of foods of vegetable origin [30].

An excessively rich diet in terms of its protein content can alter bone health. Protein intake is related to increased bone mass in childhood and adolescence [31, 32]. However, in older adults, an inverse relationship has been shown between the amount of protein in the diet and bone mass [33]. A diet very rich in protein of animal origin has been related to a decrease in bone mass, especially in individuals with poor calcium intake [34]. Hyperproteinemia would have a metabolic acidosis as a mechanism of action, which in turn would cause greater bone resorption and, as a consequence, hypercalciuria and/or an increase in glomerular filtration of calcium at the renal level [26].

In our study, we have found a negative correlation between the values of δ^15^N and the radiological indexes of bone mass. These data corroborate the first finding of this study, dealing with a rural population with a normal BMD far removed from a hyperproteic diet. The consumption of cheese, cottage cheese, eggs, and meat (protein diet), in older adults, would cause metabolic acidosis, bone resorption, hypercalcemia with hypercalciuria, and an effect on their bone mass [26]. In our study, one woman and three adult men, who presented the highest values of this isotope, corresponded to a possible diagnosis of osteoporosis according to the radiomorphometric indices. A prominent aspect in the Muslim world, which could explain these cases of possible osteoporosis, is the ingestion of vegetables rich in oxalates (spinach, artichokes, cabbage) [35], which would induce by chelation, a decrease in the intestinal absorption of calcium and therefore, hypocalcemia, secondarily the parathormone (PTH) would be activated, with bone resorption and corresponding decrease in bone mass. Added to a possible deficit in physical activity, we would have the entire pathophysiological mechanism that could justify the changes in bone mass density observed in some individuals in this study.

The limitations of this study are the lack of quantification of both isotopes in the current population and the use of densitometry as a BMD quantification technique, the most reliable method. However, our study has put into practice a method, already used for screening for osteoporosis and osteopenia, which constitutes an original and inexpensive methodological advance for use in physical anthropology.

In conclusion, the bone mass density in mandibles belonging to two diachronic populations compared and determined by two quantitative radiomorphometric indexes is similar. Within the medieval population, there is an inverse correlation between the δ^15^N value and bone mass density, this would indicate a hypoproteic diet without repercussions on BMD.

## Data Availability

The data that support the findings of this study are available on request from the corresponding author, CBR. The data are not publicly available due to containing information that could compromise the privacy of research participants.
